# Heightened Levels of Antimicrobial Response Factors in Patients With Rheumatoid Arthritis

**DOI:** 10.3389/fimmu.2020.00427

**Published:** 2020-03-20

**Authors:** Prathapan Ayyappan, Robert Z. Harms, Jennifer A. Seifert, Elizabeth A. Bemis, Marie L. Feser, Kevin D. Deane, M. Kristen Demoruelle, Ted R. Mikuls, V. Michael Holers, Nora E. Sarvetnick

**Affiliations:** ^1^Department of Surgery-Transplant, University of Nebraska Medical Center, Omaha, NE, United States; ^2^Division of Rheumatology, University of Colorado-Denver, Aurora, CO, United States; ^3^Department of Epidemiology, Colorado School of Public Health, University of Colorado Anschutz Medical Campus, Aurora, CO, United States; ^4^Division of Rheumatology, University of Nebraska Medical Center, Omaha, NE, United States; ^5^Mary and Dick Holland Regenerative Medicine Program, University of Nebraska Medical Center, Omaha, NE, United States

**Keywords:** rheumatoid arthritis, antimicrobial proteins, EndoCAbs, sCD14, CXCL16, lysozyme

## Abstract

Rheumatoid arthritis (RA) is a chronic progressive autoimmune disease leading to considerable disability over time. The disease can be characterized by the presence of multiple autoantibodies in the serum and synovial fluid. Microbial dysbiosis is proposed to play a role in the pathogenesis of RA. Increased systemic bacterial exposure leads to elevated levels of antimicrobial response factors (ARFs) in the circulation. In the present study, we tested whether RA patients have increased levels of ARFs by analyzing the levels of multiple ARFs in serum from RA patients and healthy age and sex-matched controls. The levels of soluble CD14 (sCD14), lysozyme, and CXCL16 were significantly elevated in RA patients compared to healthy controls. Lipopolysaccharide binding protein (LBP) levels remained unchanged in RA patients compared to healthy controls. A positive correlation of LBP with rheumatoid factor (RF) was also found in RA subjects. Interestingly, the levels of anti-endotoxin core antibodies (EndoCAb) IgM, total IgM, EndoCAb IgA, and total IgA were significantly elevated in RA patients compared to healthy controls. No significant changes in the levels of EndoCAb IgG and total IgG were observed in RA patients compared to healthy controls. Furthermore, lysozyme and CXCL16 levels were positively correlated with disease severity among RA subjects. Increases in the levels of several ARFs and their correlations with clinical indices suggest systemic microbial exposure in the RA cohort. Modulation of microbial exposure may play an important role in disease pathogenesis in individuals with RA.

## Introduction

Rheumatoid arthritis (RA) is a chronic progressive autoimmune disease leading to severe disability. Genetic, environmental, and epigenetic factors instigate the production of autoantibodies and the loss of tissue tolerance in RA (1–4). These autoantibodies recognize cartilage components, cellular chaperonins, IgG molecules, and citrullinated proteins (5). Similar to other autoimmune diseases, the disease predominantly occurs in females (4, 6, 7). The disease perturbs the synovial joint lining, which undergoes hyperplasia and inflammation leading to irreversible destruction of articular cartilage, ligaments, and bone (8–10). Frequent involvement of extra-articular tissues including the heart, lungs, skin, eyes, and nervous system is associated with very high levels of autoantibodies and circulating immune complexes (11, 12). Early diagnosis can greatly improve the outcome of RA, but the disease prediction remains a challenge (4).

Recently it was hypothesized that microbial dysbiosis plays a role in the pathogenesis of RA (3, 13–16). Patients with classified RA showed alterations in the gut microbiome with a relative increase in the abundance of *Prevotella copri* and decrease in *Haemophilus* spp. compared to healthy controls (14, 17). Alterations in lung microbiota, including increased levels of members of *Pseudonocardia* suggest that distal airway dysbiosis is also associated with RA (18). A pathogenic role for *Porphyromonas gingivalis*, an oral commensal was also reported (19, 20). These changes in the gut, oral and lung microbiome could cause the leakage of bacterial products into circulation, promoting inflammation and aggravating disease (20–24).

Constant exposure of microbes in the circulation elicits an antibody response to bacteria and thus can act as a measure of microbial exposure (25). Antibodies directed against multiple bacteria have been found to be elevated in RA patients. For example, circulating antibodies directed against the periodontal bacteria *Prevotella intermedia, P. gingivalis*, and *Bacteroides forsythus* were reported (19, 26, 27). Elevated levels of IgA and IgM antibodies directed against *Proteus mirabilis* were also found in RA patients and were positively correlated with total IgA and total IgM levels (28). Antibodies against members of *Enterobacteriaceae* and bacterial nucleic acids from *P. gingivalis* and *P. copri* were detected in synovial fluid from RA patients (15, 29–31). A role of *Aggregatibacter actinomycetemcomitans* as a factor in the pathogenesis of RA has also been proposed (32, 33). Persistence of microbial products and elevated levels of antimicrobial antibodies in RA patients further suggests the role of systemic bacterial exposure in the pathogenesis and progression of the disease.

In response to microbial exposure, antimicrobial response factors (ARFs) are released into the circulation to neutralize microbial products. ARFs are diverse pleiotropic molecules that include cytokines, chemokines, anti-endotoxin core antibodies (EndoCAb), peptides, and proteases (34, 35). The bactericidal activity of many ARFs is based on their ability to disrupt the bacterial cell envelope, opsonize targets, and/or inhibit intracellular functions of bacteria. The bacterial functions disrupted by ARFs include respiration, enzyme activation, and protein and nucleic acid synthesis. ARFs also modulate immune responses. For example, ARFs can activate innate immunity by recruiting and/or activating immune cells. Furthermore, some ARFs can regulate Toll-like receptor (TLR) recognition of microbial products (36). These immunomodulatory ARFs can lead to inflammation and tissue damage in the host (37).

In the present study, we tested whether RA patients have increased levels of ARFs by analyzing the levels of multiple ARFs in serum from RA patients and healthy age- and sex-matched controls. Increased levels of ARFs may indicate an increase in systemic bacterial exposure. The ARFs tested include soluble CD14 (sCD14), lipopolysaccharide-binding protein (LBP), lysozyme, CXCL16, EndoCAb IgG, EndoCAb IgA, and EndoCAb IgM. Our results revealed a marked elevation of several ARFs in RA patients. These significant elevations of ARFs may be clinically relevant since they correlate with clinical indices. Our results point to systemic microbial exposure as a common stimulus in RA, which could perpetuate the disease.

## Materials and Methods

### Study Subjects

Subjects were recruited for the Studies of the Etiology of Rheumatoid Arthritis (SERA), a prospective longitudinal study designed to evaluate the contributions of environmental and genetic factors to the development of RA. Recruitment of RA population has been described in detail previously (38). Healthy control subjects included in this study were recruited via local advertisement from the general population and tested negative for RA related autoantibodies at their baseline visit. For both the RA and healthy control populations, the base line visit was selected for this study and the duration of the study entry would be time=0 since this was their first visit. Ethical approval for this study was obtained from University of Colorado's Institutional Review Board (COMIRB#01-675) in compliance with Declaration of Helsinki. Informed consents were obtained from each participant prior to including them in the study. Our study included 50 RA subjects (39 females and 11 males), all fulfilling the revised criteria of 1987 American Rheumatism Association (39) and 50 age- and sex-matched healthy control subjects. All but three of the RA subjects reported being currently or previously on immunosuppressive and/or immunomodulatory drugs at the time of their research study visit. Health assessment, pain index, and disease activity index were collected for RA group at their study visit. The health assessment disability questionnaire index (HAQ; range 0–3) is considered the benchmark for measuring the functional status in adults with RA (40). HAQ Total assesses the hierarchy of patient outcomes by analyzing activity index, disability index, and pain index collected in 100 mm visual analog scale. Demographics and smoking history were obtained by questionnaire. Patient data is provided in Table 1. Individual ARF values per patient are included in Supplementary Table 1.

**Table 1:** Demographic and descriptive characteristics of rheumatoid arthritis and control population.

**Variable**	**RA (*n* = 50)**	**Control (*n* =50)**	***p*-value**
Age (mean ± SD)	50.0 ± 14.7	49.2 ± 14.9	0.77
Female *n* (%)	39 (78.0)	39 (78.0)	1.00
Non-Hispanic White *n* (%)	33 (66.0)	38 (76.0)	0.42
Education > High School *n* (%)	33 (66.0)	46 (92.0)	0.002
Income > $40k *n* (%)	26 (52.0)	31 (62.0)	0.34
Ever smoke yes *n* (%)	18 (36.0)	14 (28.0)	0.32
High sensitivity CRP (median, IQR)	2.7, 1.0–6.6	1.0, 0.6–1.9	0.002
CCP2 (median, IQR)	87.2, 56.4–107.1	0.1, 0.02–0.6	<0.0001
RF nephelometry (median, IQR)	84.1, 33.2–292.6	10.1, 9.8–10.7	<0.0001
Disease duration years (mean ± SD)	12.44 ± 12.46	NA	NA
Current smoker yes *n* (%)	4 (8.3)	2 (4.0)	0.43
Shared epitope positive *n* (%)	37 (74.0)	22 (44.0)	0.003

*Missing data in the table: 1 participant missing age; 23 controls missing high sensitivity CRP (mg/L); 5 controls missing anti-CCP2; 5 controls missing RF nephelometry*.

*n = total number of cases/individuals in the population*.

### Sample Collection

Venous blood was drawn in BD Vacutainer^®^ serum separator tubes (Franklin Lakes, NJ, USA) from both RA patients and healthy controls. After clotting, the whole blood collected was centrifuged (for 10 minutes at 3,000 × g and 20°C) and the serum layer was removed. Measurements of rheumatoid factor (RF), high sensitivity C-reactive protein (CRP), and anti-cyclic citrullinated protein antibodies (anti-CCP) in the serum were measured using previously described methodologies (38–41). Multiple aliquots were made from all the serum samples and stored at −80°C until analysis.

### Measurement of Analytes in the Serum

sCD14 and LBP were measured using sandwich ELISA kits procured from R&D systems (Minneapolis, USA) and Hycult Biotech (Pennsylvania, USA), respectively. EndoCAb IgG, EndoCAb IgA, and EndoCAb IgM were measured using direct ELISA kits procured from Hycult Biotech. CXCL16 was analyzed using a sandwich ELISA kit procured from Thermo Scientific (Frederick, MD, USA). Lysozyme levels were measured using sandwich ELISA kit procured from MBL (Massachusetts, USA). Total IgG, IgA, and IgM were measured using sandwich ELISA kits procured from Invitrogen (Carlsbad, CA, USA). To block non-specific antibodies that may interfere with the assay, the samples were diluted in appropriate buffers, which contained 50 μg/ml of HeteroBlock (Omega Biologicals, Bozeman, MT, USA) and kept for 30 minutes before adding into the ELISA plate. All the analyses were performed blinded to case/control and clinical status. In order to maintain the test quality and reproducibility, an internal control was included in all the assays and the coefficient of variation (CV) of replicates was set at ≤ 10%.

### Statistical Analysis

We transformed all the data into base-10 logarithm values for statistical analysis and correlation studies. Square root transformation was employed for disease indices, which included true zeros. For testing statistical significance, the unpaired *t*-test was used. For correlation analysis, Pearson product-moment correlation coefficient (Pearson's r) analysis was performed. Gender stratification was also done to detect sex-related changes in the levels of ARFs in RA subjects compared to their respective control subjects. For all statistical tests, *P* < 0.05 was considered to be statistically significant. All the statistical tests were done with GraphPad Prism 7 (GraphPad Software, Inc., San Deigo, USA). Descriptive statistics of all the analyte levels are given in Supplementary Table 2.

## Results

### sCD14 Levels Are Increased in RA Patients

**Figure 1:**
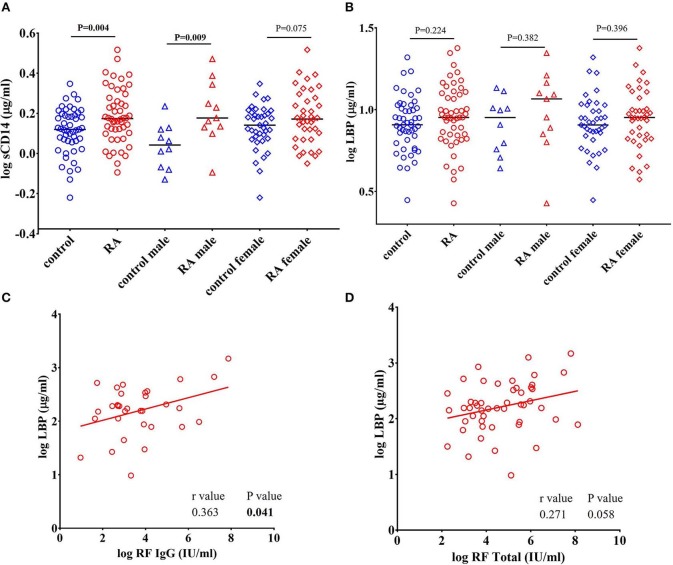
Levels of sCD14 are increased in RA patients. **(A)** Circulating levels of sCD14 in RA patients showed a significant elevation compared to healthy controls. Gender stratification revealed a significant elevation of sCD14 only in RA males compared to healthy control males while RA females showed a trend toward increase in the levels of sCD14 compared to healthy control females. **(B)** Concentration of LBP in healthy controls and RA patients. LBP levels were not significantly different in RA patients compared to healthy controls. Bars represent median analyte levels. **(C)** LBP is correlated with rheumatoid factor IgG (RF IgG). Correlation analysis revealed a significant positive correlation of LBP with RF IgG **(C)** and a trend toward significant positive correlation with total rheumatoid factor (RF Total) **(D)**.

Since CD14 acts a co-receptor for LPS, elevated levels of sCD14 are considered to reflect LPS exposure and subsequent monocyte/macrophage activation (42–44). Interestingly, Gram-positive bacterial cell wall components can also bind with CD14 (44–46). Binding of endotoxins to CD14 activates TLRs and promotes the release of proinflammatory cytokines (47, 48). We found a significant increase in the levels of sCD14 in RA patients (*P* = 0.004) compared to healthy controls (Figure 1A). Gender stratification showed that sCD14 levels in the male RA cohort were significantly elevated compared to control males (*P* = 0.009). RA females showed a trend toward an increase in sCD14 levels when compared to control females (*P* = 0.075).

### LBP Levels Positively Correlate With Rheumatoid Factor (RF) in RA Subjects

LBP is an acute phase protein synthesized by hepatocytes involved in the transfer of LPS to CD14, which partners with TLR4 expressed on innate immune cells (49, 50). We determined that LBP levels were increased in our RA cohort, however the results did not achieve statistical difference (*P* = 0.224) (Figure 1B). A positive correlation between LBP and CRP (*r* = 0.335, *P* = 0.017) in RA patients is observed in our study (Supplementary Figure 1). However, we did not find a correlation between CRP with any other ARFs that we measured.

RF was the first described autoantibody in RA and is directed against the Fc region of IgG. RF is also a valuable biomarker in terms of disease severity, diagnosis and prognosis in RA (5, 51). We found that levels of RF IgG were positively correlated with LBP (*r* = 0.363, *P* = 0.041) (Figure 1C). Furthermore, total antibody levels for all RF isotypes were also positively, though weakly, correlated with LBP (*r* = 0.271, *P* = 0.058) (Figure 1D). We did not find any correlation between RF and other ARFs that we measured (Data not shown).

### Levels of Lysozyme Are Increased in RA Patients

**Figure 2:**
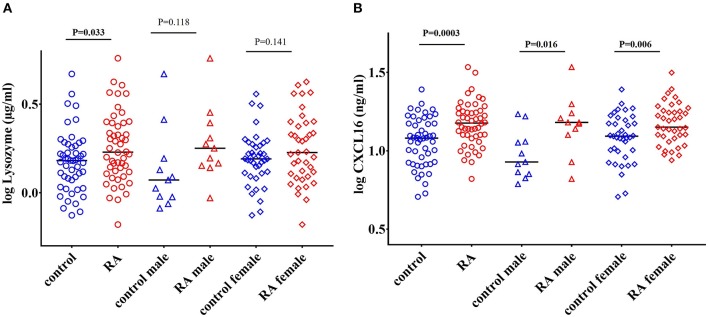
Elevated levels of lysozyme and CXCL16 in RA subjects. **(A)** Circulating levels of lysozyme are significantly elevated in RA patients compared to healthy controls. Following gender stratification, the lysozyme levels were similar in both the male and female RA cohorts compared to respective healthy controls. **(B)** RA patients have elevated levels of CXCL16 in the circulation compared to healthy controls. A significant increase in the levels of CXCL16 was also observed in both the male and female RA patients compared to respective healthy controls. Bars represent median analyte levels.

Lysozyme is an important ARF that is secreted by monocytes, macrophages, neutrophils, glandular cells, and dendritic cells. Lysozyme kills bacteria by hydrolyzing the peptidoglycan component of the bacterial cell wall. Lysozyme also possess bactericidal activity against Gram-negative bacteria (52, 53). We observed a significant increase in the levels of lysozyme in RA patients (*P* = 0.033) compared to healthy controls (Figure 2A). However, gender stratification of RA subjects showed that neither RA males (*P* = 0.118) nor RA females (*P* = 0.141) (Figure 2A) were significantly different than their respective healthy controls.

### Levels of CXCL16 Are Increased in RA Patients

CXCL16 is an important chemokine that acts as a mediator of the innate immune response (54). CXCL16 mediates adhesion and phagocytosis of both Gram-negative and Gram-positive bacteria and acts as a strong chemoattractant for CXCR6+ T cells (55–57). CXCL16 levels are also affected by alterations in the gut microbiome (58). We observed a significant elevation in the levels of CXCL16 in RA patients (*P* = 0.0003) compared to healthy controls (Figure 2B). Both RA males (*P* = 0.016) and RA females (*P* = 0.006) showed a significant increase in the levels of CXCL16 compared to their respective controls (Figure 2B).

### sCD14 Levels Positively Correlate With LBP, Lysozyme, and CXCL16 in RA Subjects and Healthy Controls

**Figure 3:**
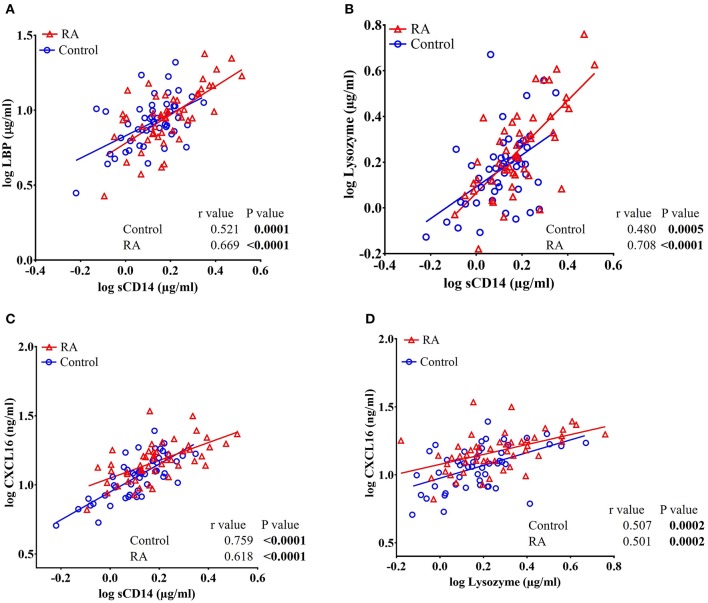
Circulating levels of sCD14 are positively and significantly correlated with LBP, lysozyme, and CXCL16 in RA patients and healthy controls. **(A–C)** Analysis showing a significant positive correlation of sCD14 with LBP, lysozyme, and CXCL16 in both RA patients and healthy controls. **(D)** Analysis showing a significant positive correlation between CXCL16 and lysozyme in RA patients and healthy controls.

Pearson's *r* analysis showed a significant positive correlation of sCD14 with LBP (*r* = 0.669, *P* < 0.0001 in RA subjects and *r* = 0.521, *P* = 0.0001 in healthy controls), lysozyme (*r* = 0.708, *P* < 0.0001 in RA subjects and *r* = 0.480, *P* = 0.0005 in healthy controls), and CXCL16 (*r* = 0.618, *P* < 0.0001 in RA subjects and *r* = 0.759, *P* < 0.0001 in healthy controls) in both RA patients and in healthy controls (Figures 3A–C). Moreover, a significant positive correlation between CXCL16 and lysozyme (*r* = 0.501, *P* = 0.0002 in RA subjects and *r* = 0.507, *P* = 0.0002 in healthy controls) was also observed in both RA subjects and in healthy controls (Figure 3D).

### Total IgA and IgM levels, Including EndoCAb-Specific IgA and IgM, Are Increased in RA Patients

**Figure 4:**
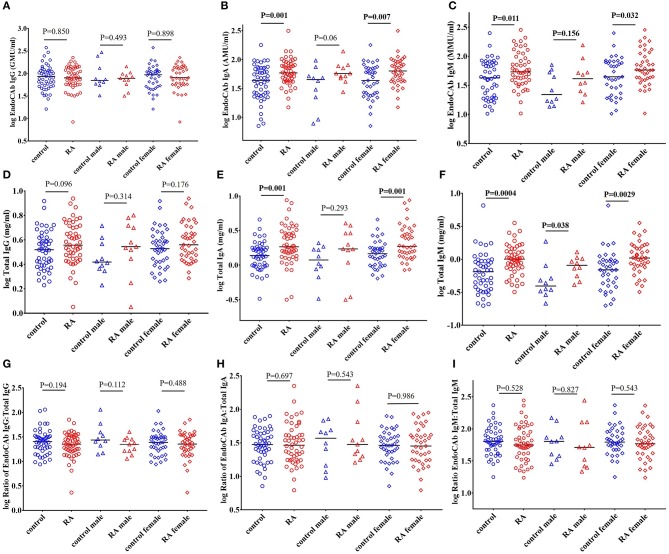
Elevated levels of total IgA and total IgM, including EndoCAb-specific IgA and IgM, in RA patients. **(A)** Circulating EndoCAb IgG levels were not significantly different in RA patients compared to healthy controls with or without gender stratification. **(B)** Circulating levels of EndoCAb IgA were significantly elevated in RA patients compared to healthy controls. RA females showed a significant elevation of EndoCAb IgA whereas RA males showed a trend toward significance compared to their respective healthy controls. **(C)** Levels of circulating EndoCAb IgM were significantly elevated in RA patients compared to healthy controls. Gender stratification revealed a significant increase of EndoCAb IgM in RA females whereas RA males did not show any significant change compared to their respective healthy controls. **(D)** Circulating levels of total IgG were similar in RA and controls, with or without gender stratification. **(E)** Total IgA levels were significantly increased in RA patients compared to healthy controls. RA females showed a significant elevation of total IgA whereas in RA males the changes did not achieve any statistical significance **(F)** Levels of circulating total IgM were significantly elevated in RA patients compared to healthy controls. Both the RA males and RA females showed a significant elevation in the levels of total IgM compared to their respective healthy controls. **(G–I)** Ratio of EndoCAbs:total Igs were not significantly different in RA patients than the healthy controls. Gender stratification also showed no significant changes in the ratios of EndoCAbs:total Igs compared to their respective healthy controls. For all figures, bars represent median analyte levels.

EndoCAbs are antibodies directed against the endotoxin core of LPS. They bind and neutralize LPS activity (59–61). We did not find significant changes in the levels of EndoCAb IgG in RA subjects compared to healthy controls (Figure 4A). Interestingly, levels of EndoCAb IgA (*P* = 0.001) and EndoCAb IgM (*P* = 0.011) were elevated in our RA cohort compared to healthy controls (Figures 4B,C). RA females showed a significant increase in the levels of EndoCAb IgA compared to control females (*P* = 0.007) whereas males showed only a trend toward an increase (*P* = 0.06) (Figure 4B). EndoCAb IgM levels were found to be significantly elevated in RA females (*P* = 0.032) compared to control females. RA males did not show any significant difference in EndoCAb IgM levels (*P* = 0.156) compared to control males (Figure 4C).

Analysis of total immunoglobulins (Igs) in RA cohort showed that IgA (*P* = 0.001) and IgM (*P* = 0.0004) were significantly elevated in RA patients compared to healthy controls, confirming earlier reports (62–64). The levels of IgG were also elevated in the RA cohort but did not achieve statistical significance (*P* = 0.096) (Figure 4D). RA females showed a significant elevation of IgA (*P* = 0.001) whereas RA males showed a trend toward significant increase (*P* = 0.293). IgM levels of both RA males (*P* = 0.038) and RA females (*P* = 0.003) were elevated compared to respective control subjects (Figures 4E,F).

We then assessed whether the proportion of EndoCAbs was elevated by analyzing the ratios of EndoCAbs:total Igs in all the groups. We did not find any significant difference between the ratios of EndoCAb IgG:total IgG (*P* = 0.194), EndoCAb IgA:total IgA (*P* = 0.697), and EndoCAb IgM:total IgM (*P* = 0.528) in RA subjects compared to healthy controls (Figures 4G–I).

### Correlations of EndoCAbs With sCD14, and Lysozyme in RA Subjects

**Figure 5:**
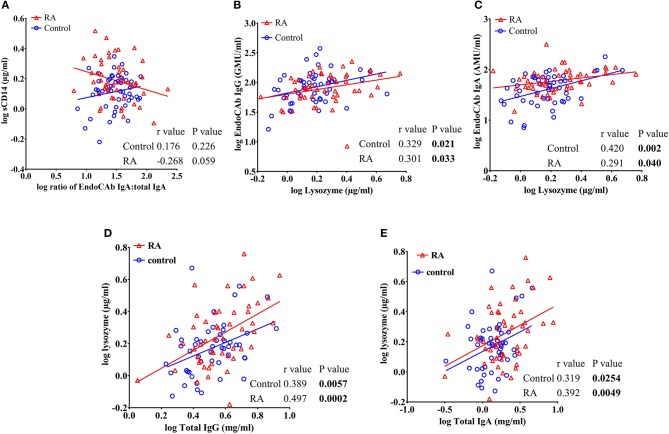
sCD14 levels correlate with the ratio of EndoCAb IgA:total IgA in RA patients. **(A)** Analysis showing a trend toward negative correlation between sCD14 and the ratio of EndoCAb IgA:total IgA in RA patients whereas healthy controls did not show any significant correlation between these values. **(B–E)** Analysis showing a significant positive correlation of circulating levels of lysozyme with EndoCAb IgG, EndoCAb IgA, total IgG, and total IgA in both RA patients and healthy controls.

In order to determine whether the levels of EndoCAbs were associated with other factors, we analyzed the correlations of EndoCAbs with other ARFs and RF. We observed a negative correlation between the ratio of EndoCAb IgA:total IgA with sCD14 in RA subjects (*r* = −0.268, *P* = 0.059). Healthy controls did not show any significant correlation between these factors (*r* = 0.176, *P* = 0.226) (Figure 5A). We found a significant positive correlation between lysozyme and EndoCAb IgG (*r* = 0.301, *P* = 0.033 in RA subjects; *r* = 0.329, *P* = 0.021 in healthy controls) and EndoCAb IgA (*r* = 0.291, *P* = 0.040 in RA subjects; *r* = 0.420. *P* = 0.002 in healthy controls) in both RA subjects and healthy controls (Figures 5B,C). Moreover, lysozyme levels were also found to be positively correlated with total IgG (*r* = 0.497, *P* = 0.0002 in RA subjects; *r* = 0.389, *P* = 0.0057 in healthy controls) and total IgA levels (*r* = 0.392, *P* = 0.0049 in RA subjects; *r* = 0.319, *P* = 0.025 in healthy controls) in both RA patients and healthy controls (Figures 5D,E).

### Lysozyme Levels Positively Correlate With Total Health Assessment Disability Questionnaire Index (HAQ Total) Values in RA Subjects

**Figure 6:**
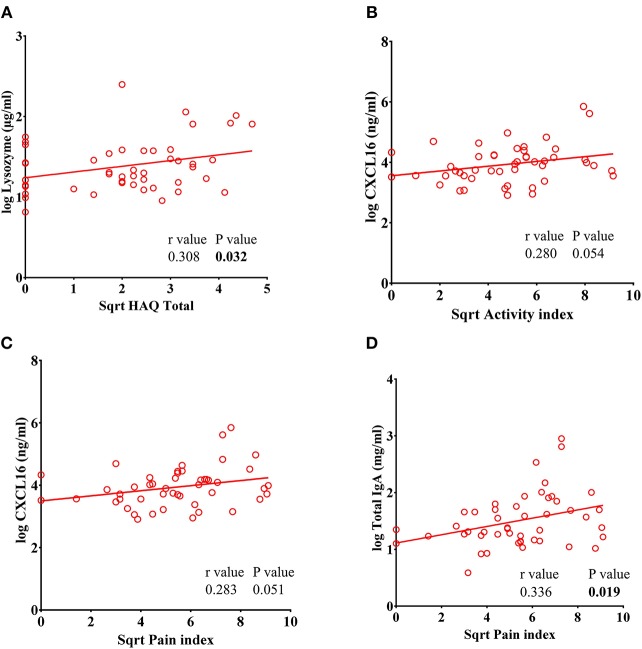
Lysozyme levels correlate with Total Health Assessment Quality questionnaire disability index (HAQ Total) in RA patients. **(A)** Analysis showing a significant positive correlation of lysozyme with HAQ total in RA patients. **(B–C)** CXCL16 levels showed a trend toward significant positive correlation of CXCL16 with pain index and disease activity index in RA patients. **(D)** Analysis showing a significant positive correlation between pain index and total IgA levels in RA patients.

We found a significant positive correlation between lysozyme levels and the HAQ Total index (*r* = 0.308, *P* = 0.032) (Figure 6A). A trend toward a positive correlation was observed in the pain index vs. CXCL16 (*r* = 0.280, *P* = 0.051) and the activity index vs. CXCL16 (*r* = 0.283, *P* = 0.054) in RA subjects (Figures 6B,C). Furthermore, a significant positive correlation between pain index and total IgA was also observed (*r* = 0.336, *P* = 0.019) (Figure 6D). Thus, the levels of these analytes parallel clinical measurements of disease severity.

No other ARFs showed a significant association with disease activity measures. In addition, we did not find any significant correlation between the levels of anti-CCP antibodies with ARFs in RA patients (data not shown).

## Discussion

Systemic exposure to microbial products has been hypothesized to trigger and/or potentiate several autoimmune diseases including RA (65–67). In response to microbial products, multiple ARFs are released into circulation as a protective mechanism to clear microbes and reduce inflammation (68, 69). Interestingly, increased circulatory levels of several ARFs in response to bacterial infection such as in sepsis also indicates that these ARFs may be specific for infection (70–73). In this study, we found increased levels of multiple ARFs in RA patients.

We observed a significant elevation of sCD14 levels in RA patients compared to healthy controls confirming previous reports (74–76). sCD14 acts as a co-receptor for endotoxin and facilitates the activation of those cells which are devoid of membrane bound CD14 via TLR4 transmembrane signaling (77–80). Elevated levels of sCD14 could be caused by bacterial exposure, alterations in the microbiome, compromised gut integrity and increased levels of cytokines. This would induce monocyte/macrophage activation and elevate the circulating concentrations of sCD14 (43, 81–86). Release of sCD14 by synovial macrophages was also suggested to contribute to elevated levels in RA patients (74). Overproduction of sCD14 by macrophages may act as a death associated molecular pattern (DAMP) and induce the production of proinflammatory cytokines (87). Thus, the elevated levels of sCD14 in circulation contributes to the maintenance of tissue inflammation by increasing the responsiveness against endotoxins (82, 87). Alternatively, elevated levels of sCD14 were reported to reduce the interaction between LPS and monocytes thereby reducing the adverse effects of monocyte/macrophage activation (42, 88). Due to the ambiguities in these experimental outcomes, further investigations are required to define the pathophysiological role of elevated levels of sCD14 in the circulation.

Similar to sCD14, LBP is a critical circulatory molecule involved in endotoxin clearance (89). We did not observe any significant difference in the levels of LBP in RA subjects compared to healthy controls. Our findings are in contrast to a recent report of elevated levels of LBP in RA patients (90). From their results, it is proposed that LBP is a specific and sensitive biomarker for RA (90). The discrepancies with our results could reflect differences in assay methodology or patient populations. Regarding methodology, while the assays are identical, the inclusion of HeteroBlock in our study could make a difference. Autoantibodies produced in RA such as RF cause interference in some immunoassays (91). RF can generate false signals by bridging capture and detection antibodies in sandwich ELISAs (92), an effect that can be mitigated through HeteroBlock (91, 93). A lack of corrective measures to block the RF interference in the study reported by Wen et al. (90) could explain their results. Differences in the two patient populations could also affect the observed differences in LBP levels. It should be noted that the LBP values for RA subjects reported by Wen et al. (90) were comparable with those reported for sepsis and other severe illnesses (73, 94, 95). Furthermore, their reported CRP values were substantially higher than those for our cohort.

Levels of lysozyme were significantly elevated in RA patients, confirming previous observations (96). Lysozyme is an important bacteriolytic enzyme produced by monocytes, macrophages, neutrophils, dendritic cells and glandular cells (52, 97). The antimicrobial potential of lysozyme is derived from its ability to hydrolyze the glycosidic bond of peptidoglycan, which is found in the cell walls of both Gram-positive and Gram-negative bacteria (94). In circulation, lysozyme facilitates the degradation of bacterial peptidoglycan into peptidoglycan monomers. This leads to the activation of myeloid cells via various pattern recognition receptors (52). Bacterial exposure can elevate the levels of lysozyme by increasing the activation of monocytes/macrophages and neutrophils (97–100). Moreover, proinflammatory cytokines released by activated macrophages can elevate the production and/or release of lysozyme (96, 97, 101).

We found that CXCL16 was elevated in our RA subjects compared to healthy controls, confirming earlier results (9, 102). CXCL16 is recognized as an antimicrobial protein involved in the adhesion and phagocytosis of bacteria (54, 55, 103, 104). Moreover, CXCL16 serves as a chemoattractant that mediates the recruitment of CXCR6-expressing immune cells and mediates inflammation (105–107). The binding of LPS with CD14 triggers the activation of NF-κB, inducing the release of CXCL16 (108, 109). Elevated circulatory levels of CXCL16 in RA patients may reflect systemic inflammation. Interestingly, we also observed a positive correlation of CXCL16 with disease severity (pain index and activity index). Li and colleagues (9) also reported a significant positive correlation of CXCL16 and RA disease activity.

B cells play an important role in the pathogenesis of RA by secreting autoantibodies, presenting antigens and producing cytokines. We observed increased levels of total IgA and total IgM in RA patients compared to healthy controls. Increased levels of total IgA and IgM in RA patients reflects activation of the immune system (61–63). EndoCAbs are endotoxin core antibodies, which can bind and neutralize circulating LPS (58). Similar to total Igs, EndoCAb IgA and EndoCAb IgM levels were significantly increased in RA patients compared to healthy controls. Elevated levels of IgM and IgA specific to some bacterial species were also observed in RA patients (28, 110). However, we found that the ratios of EndoCAb Igs:total Igs were not different from controls. This indicates that elevated levels of IgA and IgM may be due to polyclonal B cell activation. Systemic exposure of microbial products leads to polyclonal B cell hyperactivation and elevated levels of Igs (111–113). From our study, it appears that monocyte/macrophage activation is the likely cause of B cell activation and subsequent increase of total Igs and EndoCAbs in RA patients. Apart from microbial products, sCD14 was found to activate B cells (114). In addition, Ig secretion could be stimulated by cytokines released by activated monocytes/macrophages and dendritic cells (115–117).

Similar to other autoimmune conditions, RA mainly affects females (6). We observed gender differences in the levels of some ARFs. Levels of sCD14 in RA males were significantly elevated compared to control males and not females. Conversely, EndoCAb IgA, EndoCAb IgM, and total IgA levels were higher in RA females compared to control females, but were unaffected in males. This could reflect gender-specific changes in the microbiome, which were found to modulate the immune response distinctly in males and females (118–120). These gender-specific changes in gut microbiota could drive gender-biased autoimmunity (121, 122).

Multiple studies suggest the potential role of microbes as triggering and/or accelerating factors in autoimmunity (66, 123–126). Our previous studies with systemic lupus erythematosus (SLE) samples showed elevated levels of ARFs similar to what we observed in this RA study. Similar to SLE, RA subjects also showed elevated levels of total IgA, sCD14, lysozyme, and CXCL16 compared to healthy controls (127). In RA, elevated levels of IgA and total IgM along with EndoCAb IgA and EndoCAb IgM in RA indicate a global immune response. Differences in the microbiome could be a determining factor in the changes in the levels of ARFs (128).

Elevated levels of ARFs in our study support the role of myeloid cell activation in disease pathogenesis, possibly via systemic microbial exposure in RA cohort (Supplementary Figure 2). The gastrointestinal and lung microbiomes could contribute to the modulation of ARF levels. Longitudinal studies in human RA subjects are required to understand the significance of these ARFs as biomarkers for future RA development. A deeper understanding of the connection between antimicrobial responses and autoimmunity in RA could help to establish therapeutic strategies for the effective disease management in highly susceptible populations.

## Data Availability Statement

All datasets generated for this study are included in the article/Supplementary Material.

## Ethics Statement

The studies involving human participants were reviewed and approved by University of Colorado's Institutional Review Board (COMIRB#01-675). The patients/participants provided their written informed consent to participate in this study.

## Author Contributions

PA: experimental design and execution, data analysis, and manuscript preparation. RH and NS: experimental design, data analysis and reviewed the manuscript. JS, EB, MF, KD, MD, TM, and VH: substantial contribution by acquiring patient samples and clinical data and manuscript revision and review. All the authors approved the final version of the manuscript.

### Conflict of Interest

The authors declare that the research was conducted in the absence of any commercial or financial relationships that could be construed as a potential conflict of interest.
