# Supplementary material for: Heightened Levels of Antimicrobial Response Factors in Patients With Rheumatoid Arthritis
Source: Front Immunol. 2020 Mar 20;11:427. doi: 10.3389/fimmu.2020.00427 (PMC7100537; doi:10.3389/fimmu.2020.00427)
Supplement: Supplementary file 1 [file Table_1.DOCX]

**Legends to supplementary figures**

**Supplementary Figure 1.** Correlation between c-reactive protein (CRP) and LBP levels in RA patients. Correlation studies showed a significant positive correlation between CRP vs. LBP in RA patients and not in healthy controls.

**Supplementary Figure 2. Overview of the heightened levels of antimicrobial response in rheumatoid arthritis (RA).** Translocation of microbial products from oral cavity, lungs and gut into systemic circulation leads to monocyte/macrophage activation. Activation of monocytes/macrophages subsequently release antimicrobial response factors such as sCD14, lysozyme and CXCL16. Moreover, lysozyme, CXCL16 and total IgA levels were positively correlated with clinical measurements of disease severity. Microbial components can also enter into the joints, elicits an immune response and increases the disease severity. Increased levels of total IgA and IgM could be due to a more ‘global’ response that could either be EndoCAb specific or represent polyconal B cell activation. Monocyte/macrophage activation is the likely reason for B cell activation since microbial products, sCD14 and the various cytokines released by activated monocytes/macrophages are found to stimulate and activate B cells. In order to query and define this question, further studies are required to get a deeper understanding of the connection between antimicrobial responses and autoimmunity in RA.
