# Supplementary figures and images for: Heightened Levels of Antimicrobial Response Factors in Patients With Rheumatoid Arthritis
Source: Front Immunol. 2020 Mar 20;11:427. doi: 10.3389/fimmu.2020.00427 (PMC7100537; doi:10.3389/fimmu.2020.00427)

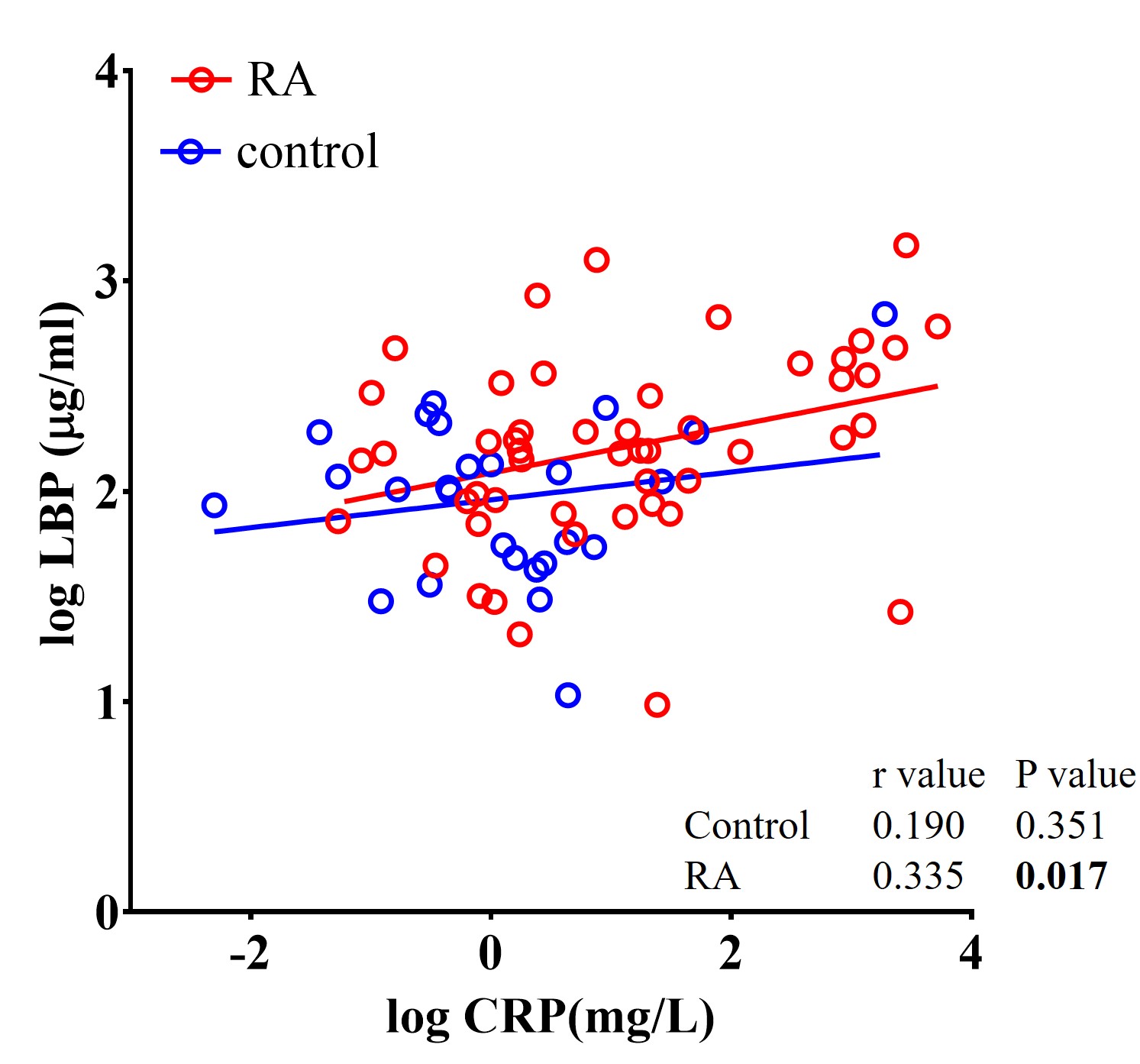

Supplement: Supplementary file 4 [file Image_1.JPEG]

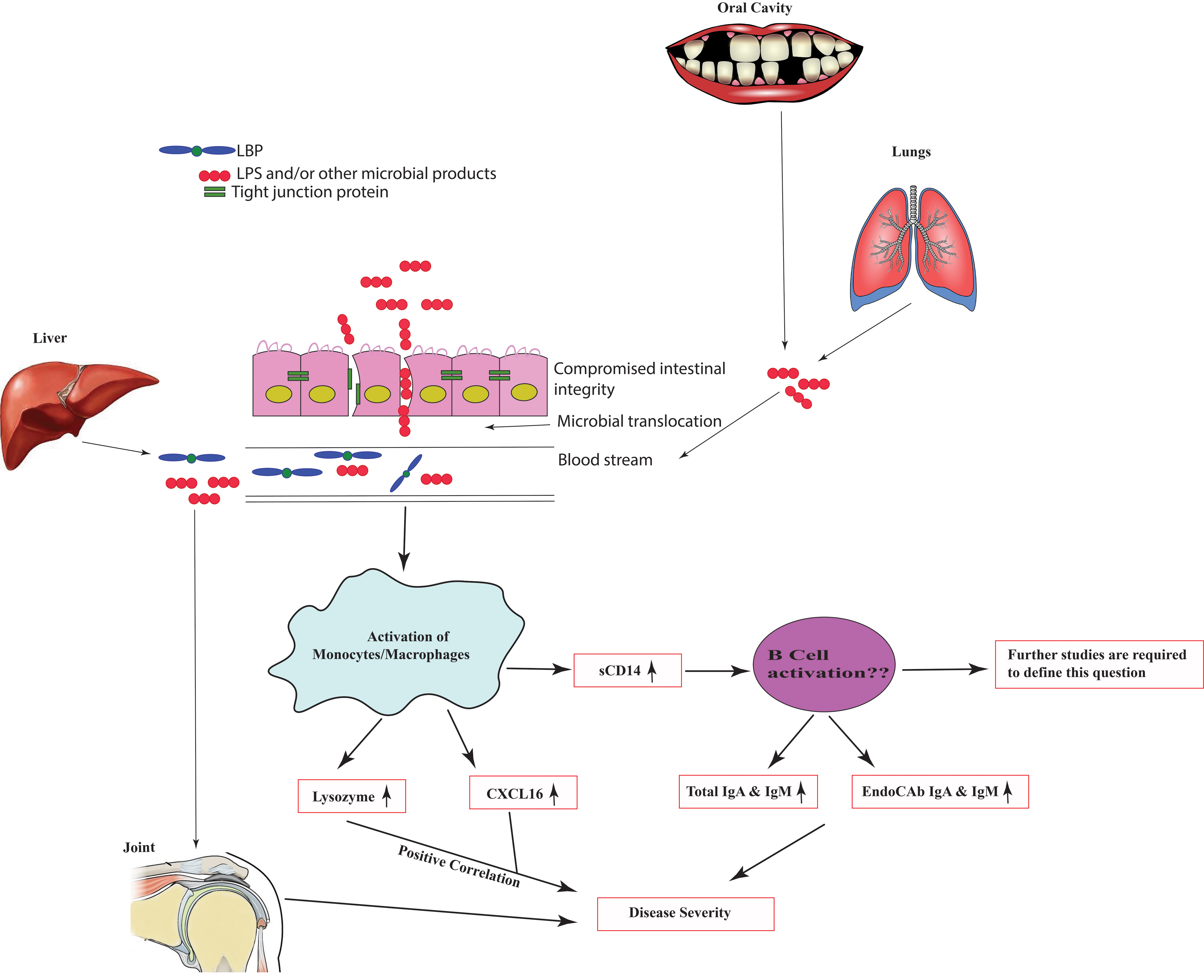

Supplement: Supplementary file 5 [file Image_2.JPEG]
